# Palmitoylation of Hedgehog proteins by Hedgehog acyltransferase: roles in signalling and disease

**DOI:** 10.1098/rsob.200414

**Published:** 2021-03-03

**Authors:** Marilyn D. Resh

**Affiliations:** Cell Biology Program, Memorial Sloan-Kettering Cancer Center, 1275 York Avenue, Box 143, New York, NY 10065, USA

**Keywords:** palmitoylation, hedgehog acyltransferase, MBOAT, Sonic Hedgehog

## Abstract

Hedgehog acyltransferase (Hhat), a member of the membrane-bound *O*-acyltransferase (MBOAT) family, catalyses the covalent attachment of palmitate to the N-terminus of Hedgehog proteins. Palmitoylation is a post-translational modification essential for Hedgehog signalling. This review explores the mechanisms involved in Hhat acyltransferase enzymatic activity, similarities and differences between Hhat and other MBOAT enzymes, and the role of palmitoylation in Hedgehog signalling. *In vitro* and cell-based assays for Hhat activity have been developed, and residues within Hhat and Hedgehog essential for palmitoylation have been identified. In cells, Hhat promotes the transfer of palmitoyl-CoA from the cytoplasmic to the luminal side of the endoplasmic reticulum membrane, where Shh palmitoylation occurs. Palmitoylation is required for efficient delivery of secreted Hedgehog to its receptor Patched1, as well as for the deactivation of Patched1, which initiates the downstream Hedgehog signalling pathway. While Hhat loss is lethal during embryogenesis, mutations in Hhat have been linked to disease states or abnormalities in mice and humans. In adults, aberrant re-expression of Hedgehog ligands promotes tumorigenesis in an Hhat-dependent manner in a variety of different cancers, including pancreatic, breast and lung. Targeting hedgehog palmitoylation by inhibition of Hhat is thus a promising, potential intervention in human disease.

## Graphical abstract

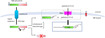

## 1. Introduction: Hedgehog synthesis and processing

Hedgehog proteins are a family of secreted, signalling proteins that regulate cell proliferation and tissue patterning during embryonic development [1]. A single protein, Hedgehog (Hh), is expressed in flies, while vertebrates express three related proteins: Sonic, Indian and Desert Hedgehog (Shh, Ihh, Dhh). Hedgehog proteins function as morphogens to generate short- and long-range signalling gradients in a concentration-dependent manner. Canonical Hedgehog signalling involves the binding of the Hedgehog ligand to its transmembrane receptor Patched1 (Ptch1), which activates the signal transducer Smoothened (Smo), leading to nuclear translocation of Gli transcription factors, and the expression of downstream target genes, including Gli1 and Ptch1 [2]. The Hedgehog pathway is mostly shut off in adults, with residual hedgehog signalling providing important support for stem cell maintenance and tissue regeneration. Aberrant over- or re-expression of Hedgehog pathway components in adult tissues is linked to tumorigenesis [3,4].

**Figure 1.**
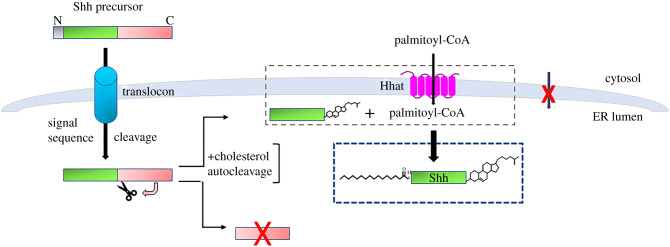
Shh biosynthesis and processing. The N-terminal signal sequence on the Shh 45 kDa precursor directs entry of Shh from the cytosol into the lumen of the ER. Following signal sequence removal, autocleavage generates a C-terminal fragment (red), which is degraded in the ER, and an N-terminal 19 kDa fragment (green). The 19 kDa Shh fragment undergoes two additional modifications. Cholesterol is attached to the C-terminus during the autocleavage reaction, and Hhat catalyses attachment of palmitate to the N-terminal cysteine. Palmitoyl-CoA, which is synthesized in the cytosol and not permeable across biological membranes, likely gains access to the ER lumen through an intramembrane tunnel within Hhat. The mature dually lipidated Shh signalling protein is indicated in the blue box.

Shh is the most extensively studied Hedgehog family member and will be used as a model to illustrate Hedgehog protein production and processing [5] (figure 1). Shh is synthesized as a 45 kDa precursor with an N-terminal signal sequence. Upon entry into the endoplasmic reticulum (ER), the signal sequence is cleaved, exposing cysteine as the N-terminal residue. Three additional processing events occur in the lumen of the ER. The C-terminal autoprocessing domain of Shh mediates autocleavage of the Shh precursor to produce two halves of the protein. The C-terminal 26 kDa portion is degraded in the ER. Concomitant with the autocleavage reaction, a molecule of cholesterol is covalently attached to the C-terminus of the 19 kDa N-terminal fragment. In a separate reaction, Hedgehog Acyltransferase (Hhat) catalyses the covalent attachment of the 16-carbon fatty acid palmitate to the N-terminal cysteine via an amide bond. The resultant, dually lipidated Shh constitutes the mature 19 kDa Shh signalling protein that is secreted from the cell.

## 2. Identification of Hedgehog acyltransferase

Using genetic screens in *Drosophila melanogaster*, four groups identified a segment polarity gene required for Hh signalling [6–9]. The gene, alternatively named Skinny Hedgehog, Sightless, Rasp or Central Missing (herein referred to as Rasp), was shown to be required for Hh activity in Hh producing cells, but was not needed for Hh production, stability, secretion or autocleavage. Based on the homology of Rasp to the membrane-bound O-acyltransferase (MBOAT) family, it was hypothesized that Rasp encodes a putative Hh acyltransferase. This supposition was supported by the finding that mutation or loss of Rasp resulted in the production of non-palmitoylated, inactive Hh [6]. In humans, the Rasp orthologue is Hedgehog acyltransferase (protein-cysteine N-palmitoyl transferase), a 493 amino acid protein encoded by the HHAT gene (accession: Q5VTY9.1). The human cDNA was originally identified as a novel melanoma antigen (MART-2) recognized by CD8+ T cells from tumour-infiltrating lymphocytes [10]. When compared to Hhat, the predicted protein sequence of MART-2 is missing residues 91–155, and the relationship of MART-2 to the Hedgehog pathway is unclear.

Definitive evidence that Hhat is a *bona fide* palmitoyl transferase was achieved when Hhat was purified to homogeneity and shown to mediate transfer of palmitate from palmitoyl-Coenzyme A (CoA) to the N-terminal cysteine of Shh via amide bond [11]. The reaction is catalytic and near-stoichiometric *in vitro*, and selective for palmitoylation of Shh versus other palmitoylated proteins (PSD95, G*α*i, H-Ras, Wnt3A, Wnt7A). A Shh mutant defective in autocleavage and cholesterol attachment is efficiently palmitoylated [11], indicating that Hhat-mediated palmitoylation is separate and distinct from the processing reactions that occur at the Shh C-terminus.

## 3. Biochemical assays and inhibitors of Hedgehog acyltransferase enzymatic activity

Hhat activity has been assayed in cells and *in vitro*. Cell-based assays to monitor Hhat-mediated Shh palmitoylation typically start with cells co-transfected with cDNAs encoding Hhat and Shh. Cells are labelled with palmitate analogues, and Shh in the cell lysate is detected using phosphorimaging or click chemistry. For example, cells expressing Hhat and Shh can be incubated with ^125^I-Iodopalmitate, a radiolabelled palmitate analogue. Shh is immunoprecipitated from cell lysates and the amount of ^125^I-palmitate incorporated into Shh is quantified by phosphorimaging after SDS-PAGE [11]. A similar strategy can be used with cells labelled with azide- or alkyne-modified palmitate, followed by click chemistry, and has been adapted to visualize palmitoylated Shh in intact cells [12]. The activity of detergent-solubilized [13] or purified Hhat [11] can be assayed *in vitro* using an N-terminal Shh peptide containing C-terminal biotin and either ^125^I-Iodopalmitoyl-CoA or alkyne or azide-labelled forms of palmitoyl-CoA. Assays that detect palmitoylation of fluorescently labelled Shh peptides by a microfluidic mobility shift [14] or changes in polarized fluorescence [15] have also been developed to monitor Hhat activity and for high-throughput analysis.

The first small-molecule inhibitors of Hhat were identified using membrane-bound Hhat in an *in vitro* Shh palmitoylation assay [16]. High-throughput screening of a chemical library composed of approximately 85 000 compounds pinpointed molecules with a bicyclic tetrahydropyridothiazole scaffold and low μM IC50s. One compound, RU-SKI 43, was selected after further orthogonal screening. RU-SKI 43 directly inhibited Shh palmitoylation by purified Hhat *in vitro* and selectively inhibited Shh palmitoylation, but not palmitoylation of H-Ras or Fyn, myristoylation of Src, or palmitoleoylation of Wnt3a in cells [16]. An additional series of dihydrothienopyridines were developed [17], and have been shown to inhibit Hhat-mediated Shh palmitoylation and signalling in cells [18,19].

## 4. Hhat enzymology and substrate recognition

Residues within Shh that are required for recognition by Hhat have been defined using full-length Shh protein, Shh peptides and GFP fusion proteins. These studies reveal that only a short region within the N-terminal Shh sequence is required for palmitoylation. Peptides containing the N-terminal 10 or 11 amino acids of the mature Shh sequence can serve as Hhat substrates *in vitro* [11,13,16]. Of note, Hhat displays an affinity for Shh peptides similar to that for the full length, 19 kDa Shh protein in *in vitro* assays [15]. In cells, the first six amino acids following the signal sequence cleavage site are sufficient for Hhat-mediated palmitoylation of a Shh–GFP fusion protein [20]. The identity and availability of the Shh N-terminal residue is important. Stoichiometric palmitoylation occurs with an N-terminal cysteine. Shh proteins or peptides with an N-terminal alanine in place of cysteine are not substrates for Hhat, but partial activity is observed with serine (see below). A free Shh N-terminus is required, as blocking the N-terminus with either a poly-histidine tag or acetylation prevents palmitoylation [11]. The residues adjacent to and downstream of the N-terminal cysteine influence palmitoylation. The mere presence of a cysteine at the N-terminal end of a heterologous sequence is not sufficient for palmitoylation. The penultimate residue, adjacent to the cysteine palmitoylation site (glycine in Shh), can be replaced with serine, alanine, proline or glutamine, but not by lysine [20]. However, a basic residue needs to be present within the first seven amino acids of the N-terminal sequence.

MBOAT enzymes are designated as *O*-acyltransferases. Both Porcn and GOAT attach fatty acids via oxyester linkage to a serine in Wnt proteins and ghrelin, respectively. By contrast, hedgehog proteins are palmitoylated via an amide bond to the amino group on the N-terminal cysteine. Shh containing an N-terminal serine, in the form of a C24S Shh mutant, can be palmitoylated by Hhat, but to lower levels compared to WT Shh. However, cysteine to serine mutants of Shh, Hh or Spitz do not serve as substrates for Rasp [20]. These biochemical differences between the mammalian and insect enzymes are reflected in differences in the *in vivo* signalling capabilities of the mutant proteins. In mice, Shh with an N-terminal serine in place of cysteine (C25S) retains residual signalling activity for Shh-mediated skeletal patterning, compared to WT Shh. It is likely that partial palmitoylation of C25S Shh is responsible for the partial signalling activity. By contrast, in flies, an N-terminal cysteine to serine mutant of Hh is inactive and does not rescue the severe segment polarity phenotype caused by loss of Hh [21,22]. Thus, Rasp and Hhat exhibit differences in substrate recognition and/or fatty acid transfer.

Analyses of Shh proteins produced in mammalian cells revealed the presence of myristate, myristoleoylate, palmitoleoylate, stearate, oleate and arachidonate, in addition to palmitate, at the Shh N-terminus [23,24]. Moreover, the distribution of fatty acid species attached to Shh can be altered by varying the amount of exogenous fatty acid added to the growth medium [24]. These findings suggest that Hhat is capable of transferring a variety of different fatty acid species to Shh in cells. To date, the only study to directly monitor the transfer of different fatty acids to Shh *in vitro* revealed that Hhat will transfer a palmitate, but not a palmitoleoylate analogue (iodo-pentadecenoyl) to Shh [25]. However, when Shh palmitoylation was monitored with radiolabelled iodopalmitoyl-CoA and purified Hhat, fatty acyl-CoAs containing 10, 12 and 14 carbon saturated fatty acids strongly reduced palmitoylation of Shh, whereas 16 : 1 and 18 : 0 fatty acyl-CoAs were less effective inhibitors [11]. It is possible that some fatty acids can bind to Hhat and be transferred to Shh, while others may bind to Hhat but cannot be transferred. This is the situation with N-myristoyl transferase (NMT), where palmitate can bind to and inhibit the enzyme, but is not transferred to NMT substrates.

All MBOAT enzymes contain an invariant histidine (H379 in human Hhat), which, when mutated, impairs fatty acyl transferase activity [26]. The conserved histidine is therefore postulated to be part of the MBOAT active site. Detailed kinetic analyses of purified H379A Hhat revealed that this mutant exhibits a twofold reduction in palmitoylation activity, primarily due to an increased *K*_m_ and decreased *V*_max_ for Shh [27]. This suggests that H379 might be involved in an interaction with the Shh protein substrate. The actual molecular mechanism of Hhat-mediated palmitoylation has not been definitively resolved. It has been suggested that the reaction proceeds in two steps: palmitate would first attach to the cysteine sulfhydryl via a thioester link, followed by an S-to-N rearrangement to generate palmitate linked via amide bond to the N-terminal amine group [5]. In this case, the cysteine -SH group would be regenerated and could be re-acylated by Hhat. Since all of the palmitate attached to Shh has been shown to be resistant to hydroxylamine, a thioesterase would be needed to release a second thioester linked palmitate. Alternatively, Hhat could catalyse the direct attachment of palmitate to the N-terminal amine via an amide linkage, analogous to the reaction catalysed by NMT. When three-dimensional structures of Hhat bound to its protein and fatty acid substrates become available, the biochemical mechanism of Shh palmitoylation should be clarified.

## 5. Hedgehog acyltransferase, membrane-bound *O*-acyltransferases and transmembrane catalysis across the endoplasmic reticulum membrane

**Figure 2.**
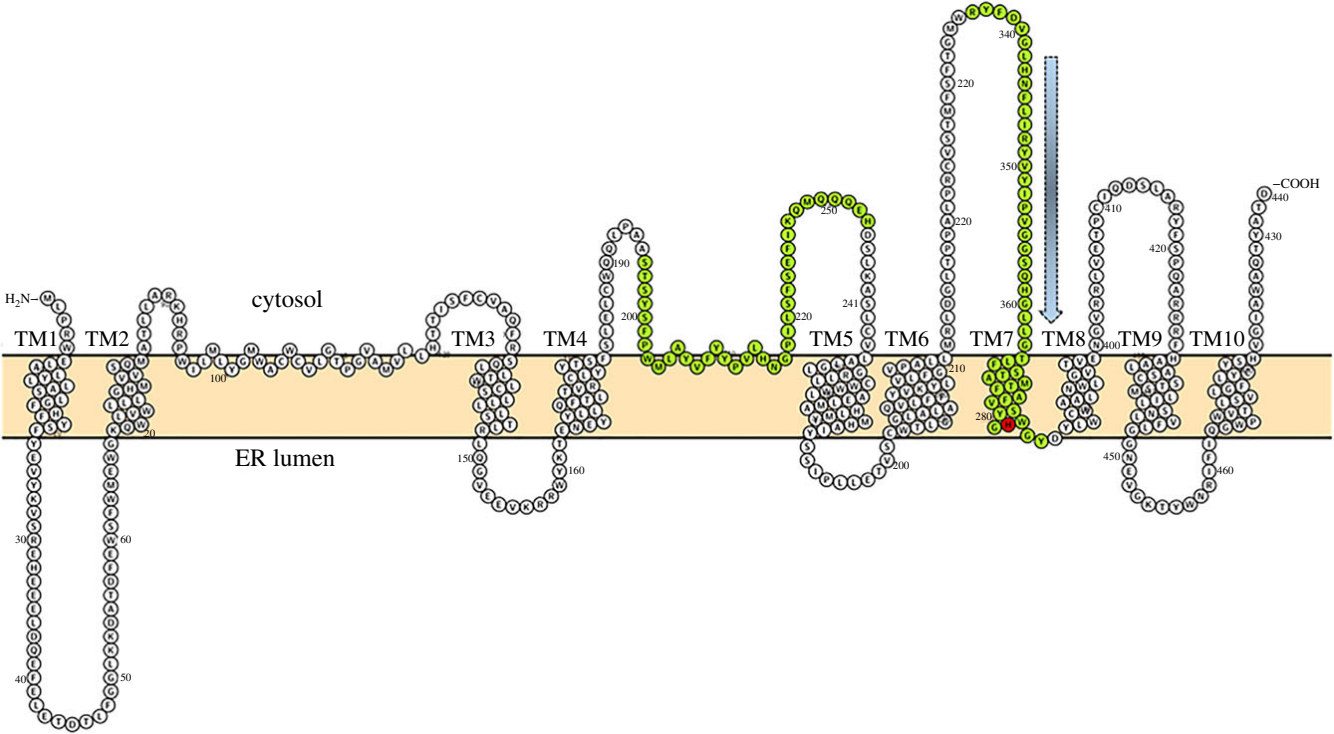
Predicted transmembrane topology map of Hhat. Hhat-predicted transmembrane domains, intramembrane loops and re-entrant loops were redrawn from data in Matevossian & Resh [28] using Protter [30]. Residues in conserved MBOAT domains, identified in [27], are highlighted in green. The conserved histidine (H379) found in all MBOATs is highlighted in red. A predicted entry point for palmitoyl-CoA into a transmembrane tunnel, based on homology to the channel in GOAT [31], is indicated with the blue arrow.

Two studies have explored the transmembrane topology of Hhat, both obtaining nearly identical predicted two-dimensional structures [28,29] (figure 2). *In silico* analysis using a variety of different membrane topology prediction programs was used to design Hhat constructs with epitope tags placed within predicted hydrophilic, loop regions. The orientation (cytoplasmic or luminal) of the tags was determined using either differential membrane permeabilization coupled with immunofluorescence microscopy in cells, or protease protection assays *in vitro*. Hhat contains ten transmembrane domains and two reentrant loops, with the N- and C-termini both in the cytosol. The conserved histidine (H379), that is found in all MBOATs and is important for catalysis, is on the luminal side of the ER membrane. The topology map of Hhat bears a striking similarity to that of GOAT, despite the fact that these two enzymes share less than 20% primary sequence identity [28]. Of note, three prominent cytoplasmic loops are present in Hhat and GOAT when the predicted topology maps of the two MBOATs are compared side-by-side. The presence of a large number of residues facing the cytoplasm suggests that these residues might be involved in protein–protein interactions, regulatory modifications or substrate binding. Of note, four cytosolic cysteine residues have been reported to be sites of S-palmitoylation on Hhat [29].

Hhat-mediated Shh palmitoylation occurs in the ER lumen [6,11], consistent with the location of H379 on the luminal side of the ER membrane. In order for the acyltransferase reaction to occur, both Hhat substrates (Shh and palmitoyl-CoA) need to access the ER lumen. The entry of Shh into the ER is promoted by interaction of its N-terminal signal peptide with the translocon in the ER membrane. A Shh construct lacking the N-terminal signal sequence remains cytosolic and is not palmitoylated. However, palmitoyl-CoA is synthesized in the cytosol and is not permeable across biological membranes. The question then becomes: how does palmitoyl-CoA reach the luminal side of the ER membrane in order for palmitoylation to occur? A recent study addressed this question by showing that Hhat itself is a conduit for palmitoyl-CoA translocation across the ER membrane [19] (figure 1). Palmitoyl-CoA uptake into the interior of microsomal membrane vesicles formed from cells overexpressing Hhat was five times higher compared to membranes from untransfected cells or from membranes from Hhat^−/−^ cells. When purified Hhat was reconstituted into large unilamellar liposomes containing a lipid composition reflective of the ER membrane, uptake of palmitoyl-CoA was detected biochemically and visualized via confocal microscopy. Treatment with a small-molecule Hhat inhibitor or mutation of H379 within Hhat inhibited palmitoyl-CoA uptake, suggesting that acyltransferase activity and palmitoyl-CoA uptake are linked and coordinated. This is supported by the finding that incorporation of a Shh peptide within the liposome slows palmitoyl-CoA uptake, while a mutant C24A Shh peptide, that cannot be palmitoylated, blocks uptake. These findings suggest that an internal tunnel within Hhat could provide a meeting place for cytosolic palmitoyl-CoA and luminal Shh and thereby serve as a site for Shh palmitoylation.

Additional structural studies support the presence of a tunnel or channel for fatty acyl-CoAs within the interior of MBOAT enzymes. The first MBOAT three-dimensional structure to be solved was that of DltB, a bacterial MBOAT that attaches D-alanine to teichoic acid in the cell wall of Gram-positive bacteria [32]. DltB contains a structural funnel that extends from the extracellular side to the middle of the membrane bilayer and is connected by a tunnel to the cytoplasmic side. The catalytic histidine sits at the base of the funnel. Cross-membrane catalysis is proposed to occur by the interaction of lipoteichoic acid bound to the funnel on the extracellular side and D-alanine, which gains access through the tunnel on the cytoplasmic side [32]. An internal channel connecting the cytoplasm and the ER lumen was also identified in a three-dimensional structure generated by computational modelling of the mammalian MBOAT GOAT [31]. The conserved histidine is located within the channel, in a position identical to that of the histidine in DltB. Transmembrane fatty acylation is proposed to occur by ‘handing off’ the octanoyl moiety from octanoyl-CoA to ghrelin within the channel. CoA would exit at the cytosolic side and octanoylated ghrelin would be released into the ER lumen [31]. The situation is similar, but slightly different in cryo-EM structures reported for DGAT1 and ACAT1, two MBOAT enzymes that catalyse the attachment of fatty acids to lipids [33–36]. Both enzymes contain a cytosolic tunnel, through which fatty acyl-CoA enters, and a large cavity within the membrane bilayer that contains the conserved histidine and serves as a reaction chamber. For DGAT1, diacylglycerol is proposed to enter the reaction chamber through a lateral gate that opens to the hydrophobic core of the lipid bilayer [33,34]. Once oleoyl is transferred to diacylglycerol, the resultant triacylglycerol product is released back into the membrane bilayer. A transmembrane or luminal leaflet tunnel also serves as the entry point for cholesterol in ACAT1, with the cholesterol-ester product released into the ER membrane [35,36]. Thus, MBOATs that transfer fatty acids to lipids (e.g. DGAT1, ACAT1) release their fatty acylated lipid products into the ER bilayer, while MBOATs that acylate proteins (Hhat, GOAT, Porcn) release their fatty acylated protein products into the ER lumen.

## 6. Requirement of palmitoylation for Hedgehog and Sonic Hedgehog signalling

Evidence for a requirement of palmitoylation has been obtained in studies of both invertebrate and vertebrate Hedgehog signalling. Growth and patterning of the *Drosophila* wing imaginal disk is abolished when a Hh mutant that cannot be palmitoylated (C85S) is expressed [21,37] or in fly embryos lacking Rasp [6]. A non-palmitoylatable Hh mutant cannot rescue the phenotype of a Hh null mutant even though it is correctly processed and expressed at levels equivalent to those of WT Hh. In the mouse limb bud and neural tube, C24S Shh has reduced patterning activity compared to WT Shh and patterning is defective in embryos lacking Hhat [21,22]. N-terminal fatty acylation of Shh enhances its ability to induce rat forebrain neuronal differentiation *in vitro*. Retroviral mediated expression of WT Shh into the developing mouse telencephalon *in vivo* induces severe brain deformities that do not occur when C24S Shh expressing viruses are injected [38]. *In vitro* assays of Shh signalling reveal that palmitoylated Shh is at least 30-fold more active compared to unacylated Shh, and that increasing the hydrophobicity of the attached fatty acid increases Shh potency [23,39]. Palmitoylation has also been shown to be required for Shh multimerization into soluble, higher molecular weight complexes that are postulated to play a role in signalling. However, the reverse is not the case, as multimerization is not required for Shh palmitoylation [22,40].

An additional function for Rasp was identified for epidermal growth factor (EGF) receptor-mediated signalling in *Drosophila melanogaster*. Rasp was shown to mediate palmitoylation of the *Drosophila* EGF ligand Spitz on its N-terminal cysteine [41]. The functional consequence is that palmitoylation restricts the movement of Spitz by increasing its local concentration at the plasma membrane of the producing cell. Two other *Drosophila* EGF receptor (EGFR) ligands, Keren and Gurken, contain an N-terminal cysteine, produced following signal sequence cleavage, and it is likely that these proteins are also palmitoylated by Rasp [41]. None of the vertebrate EGFR ligands have an N-terminal cysteine, and no Spitz/Keren/Gurken orthologues have been identified in mammals. Based on a bioinformatics-based search for potential Hhat substrates, 87 proteins in the human proteome are predicted to contain an N-terminal cysteine following the signal sequence, but other than Shh, Ihh and Dhh, none of these have been shown to be substrates for Hhat-mediated palmitoylation in mammals [20].

## 7. Sonic Hedgehog release from cells

After processing and modification in the ER lumen, dually lipidated Shh is packaged into secretory vesicles and trafficked to the plasma membrane. Release of Shh from the membrane of the producing cell requires two factors: Dispatched1 (Disp1), a multipass membrane protein that contains a sterol sensing domain, and Scube2, a soluble protein [42,43]. Disp1 and Scube2 each recognize different molecular regions of cholesterol and bind sequentially in a hand-off or relay type of mechanism to cholesterol-modified Shh. Both proteins are required for release of mature Shh from the producing cell in a cholesterol-dependent manner. However, when Shh is bound to Scube2, the palmitate-dependent interaction needed for interaction with Ptch1 is blocked (see below). Several additional factors cooperate in a molecular relay to allow the solubilized Shh to signal to recipient cells [44]. Two membrane-bound co-receptors for Shh, Cdon and Boc bind to and recruit the Scube2-Shh complex to the cell surface. Next, a third co-receptor, the GPI-anchored protein Gas1, binds the palmitate and cholesterol moieties on Shh, causing dissociation of Shh from Cdon/Boc-Scube. Gas1-bound Shh is then delivered to Ptch1 to initiate signalling.

The mechanism that enables secreted, lipidated Shh to remain solubilized so that it can traverse long distances in the extracellular space has not been fully resolved. Multiple models have been proposed, including binding to lipoprotein particles, secretion via exosomes and localization in filopodia/cytonemes [45–48]. The relative abundance of Shh modified with fatty acids other than palmitate may also influence Shh movement [24]. Regulation of the size and shape of the morphogen concentration gradient may be cell, tissue and/or organism dependent.

## 8. Role of palmitate in binding of Sonic Hedgehog to Patched1

Binding of Shh to its receptor Patched-1 (Ptch1) is the first step in the activation of Shh signalling. Ptch1 is a multipass membrane protein, containing 12 transmembrane helices (five of which include a sterol sensing domain), two extracellular domains (ECDs), and a C-terminal cytoplasmic domain. Although palmitoylation of Shh is essential for signalling, it is not required for high-affinity binding of Shh to Ptch1. Several recent studies have helped elucidate the molecular mechanism of Shh interaction with Ptch1. Biochemical analyses established that a 22-amino acid palmitoylated Shh peptide can bind to Ptch1 and activate Shh signalling; both events are dependent on Shh palmitoylation [49]. A second interaction occurs when the globular portion of Shh binds to Ptch1 at a different site. This is a high-affinity interaction that mediates Ptch1 internalization. Cryo-EM and crystallographic structures of Shh bound to Ptch in the presence of Ca++ support and extend these findings [50–52]. One Shh binds two molecules of Ptch1, which are arranged asymmetrically in a membrane-bound complex [50]. The N-terminal region of Shh binds to one of the Ptch1 molecules, with palmitate inserting into a hydrophobic cavity between subdomains of the two ECDs of Ptch1 [51]. A second interaction occurs with the globular portion of Shh binding, in a Ca^2+^-dependent manner, to the ECDs of the other Ptch1 molecule. Both interactions are apparently needed for efficient Shh signalling.

The structural studies of Shh–Ptch1 interactions also uncovered at least two binding sites for cholesterol within Ptch1 [52]. One is located in the first ECD and is occupied by the cholesterol attached to the C-terminus of Shh [53], the other is within a cavity in the sterol sensing domain. The two sites are connected by a 150 Å tunnel, which has been postulated to provide a mechanism for cholesterol transport by Ptch1 [50,52]. Insertion of the N-terminal palmitate and C-terminal cholesterol on Shh blocks the tunnel when Shh is bound to Ptch1. The following model has been proposed to explain how binding of palmitoylated Shh regulates Ptch1-dependent Shh signalling [54]. In the absence of Shh, cholesterol transport by Ptch1 depletes levels of accessible cholesterol from the plasma membrane within the primary cilia, where Ptch1 regulation of Smoothened occurs. Since Smoothened activity is dependent on cholesterol, Smoothened is kept in an inhibited state. Binding of Shh occludes the cholesterol tunnel, and inactivated Ptch1 exits the cilium. This results in the restoration of cholesterol in the membrane, the activation of Smoothened and consequent Smoothened-dependent Shh signalling.

## 9. Hedgehog acyltransferase mutations and disease physiology in humans and mice

Loss of Hhat is lethal in mice and phenocopies the mutant phenotypes observed with either loss of Shh expression or expression of non-palmitoylated mutant Shh [22]. Hhat^−/−^ mice, in which the null allele of Hhat was generated by gene targeting, die shortly after birth and exhibit holoprosencephaly and limb defects, affecting both Shh and Ihh signalling. In the Creface transgenic line of mice, in which the Cre recombinase is driven by an AP2A enhancer, the Creface transgene is integrated within exon 9 of Hhat, resulting in Hhat loss of function. Hhat^creface^ mice exhibit severe craniofacial defects, including holoprosencephaly, along with defects in cartilage and bone differentiation [55]. These phenotypes reflect defective Shh signalling.

To date, there have been several reports of HHAT mutations linked to disease states or abnormalities in humans. A biallelic missense mutation (L257P) within Hhat was identified in two siblings that exhibited brain disorders (microcephaly, early infantile-onset seizures) [56]. The mutated residue is predicted to lie within TMD5 of Hhat. A heterozygous *de novo* mutation in Hhat (W386C) was detected in two siblings from a consanguineous family that suffer from intellectual disability, but a causative role for this variant in the disease has not been defined [57]. Hhat is expressed in developing gonads at the time of sex determination, and later in Sertoli cells, which produce Dhh. Callier *et al.* [58] identified a homozygous G287 V missense mutation in Hhat in a patient with abnormal testicular development. The G287 V mutation was shown to inhibit Hhat-mediated palmitoylation of Shh and Dhh *in vitro* [58]. The absence of functional Hhat severely affects testis development, probably due to the unique role of Dhh in the testis.

## 10. Hedgehog acyltransferase and Sonic Hedgehog signalling in disease

Aberrant re-expression of and signalling by Shh in adult tissues is implicated in a variety of human disease states, particularly cancer [1,3]. This can occur via ligand-dependent and ligand-independent mechanisms. Activating mutations in Smoothened and inactivating mutations in Ptch1 lead to the constitutive activation of the Shh pathway in advanced basal cell carcinoma and medulloblastoma. In these instances, signalling is independent of Shh ligand, and inhibition of Shh palmitoylation cannot alter activated, downstream signalling. By contrast, ligand-dependent pathway activation occurs in lung, pancreatic and breast cancers, which should be susceptible to Hhat inhibition. TCGA database analysis reveals that Hhat is overexpressed in a number of human lung cancer tumours. Two studies have identified a key role for Hhat in non-small cell lung cancer (NSCLC), where Shh expression has been shown to drive proliferation and tumorigenesis. RNAi-mediated knockdown of Hhat inhibits Shh pathway activation and growth of NSCLC cells *in vitro* and tumour growth *in vivo* in mouse xenografts [59]. Approximately 70% of lung squamous cell carcinomas (LSCC), a subtype of NSCLC, exhibit amplification of chromosome 3q26. This results in overexpression of protein kinase C iota (PKC*ι*), which phosphorylates SOX2, a stem cell transcriptional regulator. When SOX2 is phosphorylated by PKC*ι* it binds to the HHAT promoter, inducing Hhat expression and the activation of Shh signalling [60]. This pathway drives LSCC cells into a cancer stem cell (CSC) phenotype. CSCs are a tumour subpopulation that can self-renew and give rise to heterogeneous lineages that repopulate the tumour. Hhat knockdown has been shown to reduce growth and tumorigenicity of CSCs formed from LSCC [60]. In addition, Hhat overexpression has been detected in stem cell spheroids formed from human tongue squamous cell carcinoma cell line. Hhat knockdown or treatment with RU-SKI 43 inhibits spheroid growth [61]. CSCs are important to target as they are notoriously resistant to chemotherapy and are associated with increasing metastasis.

Shh is a clinically relevant target in pancreatic cancer, the fourth leading cause of cancer-related deaths in the US, with a 5-year survival rate of 5%. The normal adult pancreas does not express Shh. When aberrant Shh expression occurs in the mature pancreas, it plays a critical role in promoting pancreatic cancer [62]. Shh functions in pancreatic cancer via autocrine and paracrine mechanisms using both canonical and non-canonical (Smoothened-independent) pathways. Hhat knockdown or treatment with a small-molecule inhibitor of Hhat blocks Shh autocrine and paracrine signalling and the growth of pancreatic cancer cells *in vitro* [63,64].

Shh and Hhat are implicated in the growth of breast cancer [65]. Breast tumour growth and metastasis in mice is stimulated by Shh overexpression and is decreased by inhibiting Shh. In humans, Shh overexpression occurs in breast tumour initiating cells and in invasive ductal carcinoma, where it is associated with increased metastasis and death from breast cancer. Hhat knockdown or treatment with RU-SKI 43 was shown to inhibit oestrogen receptor positive, HER2 amplified and tamoxifen-resistant breast cancer cell growth, while Hhat overexpression increased ER positive cell proliferation [66]. Smo inhibitors had no effect, suggesting that Hhat and Shh are signalling via non-canonical pathways.

Typically, Hedgehog is a negative regulator of Wnt signalling in the normal intestine. However, in colon cancer, Shh and Wnt signalling are both enhanced in colon cancer stem cells [67]. In the CSC subpopulation, Shh drives signalling and growth via an autocrine, non-canonical pathway that is dependent on Hhat and Ptch1, but Smo and Gli1 independent. RU-SKI 43 reduced survival of colon cancer stem cells, and this effect could be rescued by the addition of recombinant Shh. Likewise, Hhat knockdown inhibited Wnt signalling and reduced the growth of CSC spheroids *in vitro* and tumour formation *in vivo* [67]. Non-canonical Hhat-dependent Hedgehog signalling was proposed to act as a positive regulator of WNT to maintain CSCs in an undifferentiated state. These findings suggest that Hhat inhibition could potentially be used to induce CSC differentiation and prevent CSCs from driving tumour growth.

Overactivation of the Hedgehog pathway has been shown to be involved in rheumatologic diseases. One example is systemic sclerosis (SSc), a disease characterized by fibrosis of the connective tissue. Fibroblasts, endothelial cells and keratinocytes from patients with SSc exhibit activated Shh signalling, and overexpression of Shh in the skin was shown to induce skin fibrosis in mice [68]. The overexpression of Hhat was detected in skin fibroblasts from patients with SSc, compared to controls. Hhat knockdown reduced Hedgehog signalling and collagen release from these fibroblasts *in vitro*, as well as skin fibrosis in the skin of mice [69]. Ihh plays key roles in the development of bone and cartilage and has been shown to be upregulated in osteoarthritis [70]. Thus, Hhat might be a potential target for inhibition in this common, painful joint disorder.

## 11. Remaining questions and future directions

Structural studies of MBOAT family members have contributed to a greater understanding of fatty acyl transferase enzymology in general. However, in the absence of a three-dimensional structure and more detailed kinetic studies, key details of Hhat enzymology remain unknown. Unlike DHHC palmitoyl transferases, an acyl-enzyme intermediate for Hhat has not been detected, nor is it known whether palmitate is directly transferred by Hhat to the N-terminal cysteine of Shh or via an S-to-N shift from acylated Shh. Based on palmitoyl-CoA uptake assays and analogy to other MBOATs, it is likely that a tunnel or channel exists within Hhat to allow transfer of palmitate from the cytoplasmic to the luminal side of the ER membrane for attachment to Shh either within the channel or near the luminal side of the membrane. In cells where Hhat is expressed but Shh is not, an ‘open’ conformation of the Hhat tunnel could potentially allow palmitoyl-CoA or palmitate to transit through the ER membrane to supply fatty acid or fatty acyl-CoA to the ER lumen. Moreover, an intramembrane tunnel within Hhat could provide a binding site for small-molecule inhibitors that inhibit Hhat catalytic activity and/or palmitoyl-CoA uptake. The geometry of the tunnel probably dictates the fatty acid substrate preference of Hhat, the resultant differentially fatty acylated species of Shh and the formation of the morphogenetic gradient for signalling in Shh recipient cells.

While Hedgehog signalling during development primarily proceeds via the canonical Ptch1/Smoothened/Gli pathway, there is increasing evidence that non-canonical, Smoothened-independent signalling pathways are operative in adult tissues and stem cell subpopulations. In this case, Smoothened inhibitors that are currently in clinical trials may not be effective. An alternative is to target upstream, at the level of Shh ligand. A number of small-molecule inhibitors of Hhat have been reported, and these compounds have shown efficacy *in vitro* and in cell-based assays. The challenge, as with all pharmacological approaches, is to establish an on-target mechanism for a particular compound in specific cell and tissue types, and this task should be aided as our knowledge of non-canonical Shh signalling pathways continues to grow. Ultimately, targeting production of palmitoylated Shh via Hhat inhibitors has the potential to provide therapeutic benefit in Shh ligand-driven human diseases.

## Data accessibility

This article does not contain any additional data.
